# Alternative splicing controls pan-neuronal homeobox gene expression

**DOI:** 10.1101/gad.352184.124

**Published:** 2025-02-01

**Authors:** Eduardo Leyva-Díaz, Michael Cesar, Karinna Pe, José Ignacio Jordá-Llorens, Jessica Valdivia, Oliver Hobert

**Affiliations:** 1 Howard Hughes Medical Institute, Department of Biological Sciences, Columbia University, New York, New York 10025, USA;; 2 Department of Developmental Neurobiology, Instituto de Neurociencias (Consejo Superior de Investigaciones Científicas [CSIC]-Universidad Miguel Hernández [UMH]), 03550 Sant Joan d'Alacant, Spain

**Keywords:** *C. elegans*, alternative splicing, homeobox, neuronal cell fate, pan-neuronal gene expression

## Abstract

The pan-neuronally expressed and phylogenetically conserved CUT homeobox gene *ceh-44/CUX* orchestrates pan-neuronal gene expression throughout the nervous system of *Caenorhabditis elegans.* As in many other species, including humans, *ceh-44/CUX* is encoded by a complex locus that also codes for a Golgi-localized protein, called CASP (Cux1 alternatively spliced product) in humans and CONE-1 (“CASP of nematodes”) in *C. elegans*. How gene expression from this complex locus is controlled—and, in *C. elegans*, directed to all cells of the nervous system—has not been investigated. We show here that pan-neuronal expression of CEH-44/CUX is controlled by a pan-neuronal RNA splicing factor, UNC-75, the *C. elegans* homolog of vertebrate CELF proteins. During embryogenesis, the *cone-1&ceh-44* locus exclusively produces the Golgi-localized CONE-1/CASP protein in all tissues, but upon the onset of postmitotic terminal differentiation of neurons, UNC-75/CELF induces the production of the alternative CEH-44/CUX CUT homeobox gene-encoding transcript exclusively in the nervous system. Hence, UNC-75/CELF-mediated alternative splicing not only directs pan-neuronal gene expression but also excludes a phylogenetically deeply conserved golgin from the nervous system, paralleling surprising spatial specificities of another golgin that we describe here as well. Our findings provide novel insights into how all cells in a nervous system acquire pan-neuronal identity features and reveal unanticipated cellular specificities in Golgi apparatus composition.

## Abstract (precis)

In this study, Leyva-Díaz et al. show that the RNA splicing factor UNC-75/CELF controls the alternative splicing and expression of two distinct transcripts from a shared, complex genomic locus in *C. elegans*—that of the nuclear CUT homeobox gene CEH44/CUX and Golgi protein CONE1. This regulatory mechanism is specifically directed toward cells of the nervous system and orchestrates pan-neuronal identity as well as cellular and temporal specificities in the Golgi apparatus.

The underlying basis for the uniqueness of any animal's nervous system is the enormous diversity of cell types that populate this organ. Neuronal gene expression programs can be broken down into two separate, parallel acting routines: (1) neuron type-specific and (2) pan-neuronal programs. Genes that are selectively expressed in specific types of neurons are coordinately controlled by combinations of master regulatory transcription factors, called terminal selectors (Hobert 2016). Loss of terminal selectors therefore results in the loss of the cell type-specific identity of a neuron class. In contrast, the loss of terminal selectors does not affect the expression of pan-neuronal genes (e.g., neuropeptide processing machinery, synaptic vesicle proteins, etc.), demonstrating the existence of parallel gene regulatory programs that define the pan-neuronal features of a neuron (Hobert et al. 2010).

We have recently uncovered the identity of pan-neuronal gene expression regulators. We found that an entire subfamily of homeobox genes, the CUT homeobox genes, operates in a redundant and dosage-dependent manner to control pan-neuronal gene expression (Leyva-Díaz and Hobert 2022). Several of the CUT homeobox genes are ubiquitously expressed, but two of them, *ceh-44/CUX* and *ceh-48/ONECUT*, are restricted to the nervous system, apparently increasing CUT gene dosage above a level sufficient to control pan-neuronal target gene expression. However, how *ceh-44/CUX* and *ceh-48/ONECUT* expression is restricted to the nervous system is not known. Here, we describe an unanticipated regulatory strategy that drives expression of *ceh-44/CUX* in the nervous system. This regulatory strategy also sheds light on a highly unusual and mysterious gene locus that is present throughout the animal kingdom, from worms to humans (Burglin and Cassata 2002; Takatori and Saiga 2008).

More than 20 years ago, in a search for proteins involved in trafficking at the Golgi apparatus, Gillingham et al. (2002) noted that a phylogenetically deeply conserved, Golgi-localized protein is encoded by a genetic locus that is also capable of producing a homeodomain protein (Fig. 1A; Supplemental Fig. S1). The Golgi-localized isoform of this locus, called CASP (Cux1 alternatively spliced product), is conserved from yeast to mammals and functions as a so-called “golgin,” a group of Golgi membrane proteins unrelated by primary sequence and involved in vesicle transport along the Golgi apparatus (Fig. 2A; Munro 2011; Lowe 2019). The homeobox-containing isoform of this locus also encodes a deeply conserved protein, the CUT homeodomain protein CUX1, the vertebrate ortholog of the *Caenorhabditis elegans* CEH-44 protein. The structural organization of this locus is such that CASP and CUX1 are predicted to share their N-terminal region, while their specific functional domains are located to the C-terminal part of the respective proteins (Fig. 1A).

**Figure 1.**
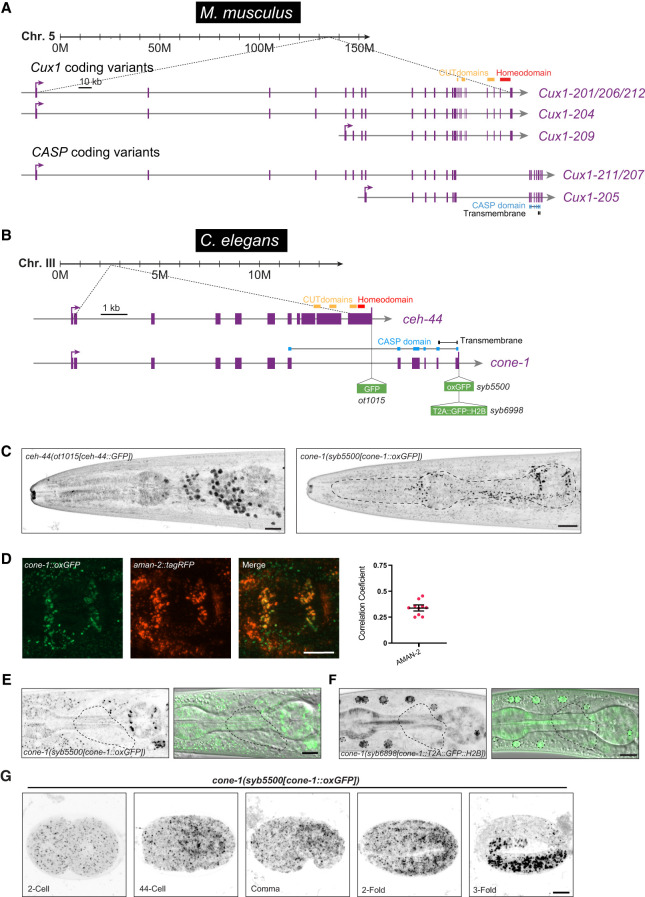
*cone-1&ceh-44* locus structure and expression pattern. (*A*) Schematic illustration of the mouse *Cux1* locus showing the predicted transcripts coding for Cux1 and CASP (Ensembl release 112; Harrison et al. 2024), as well as the locations of the homeodomain and CUT, CASP, and transmembrane domains. (*B*) Schematic representation of the *C. elegans cone-1&ceh-44* gene locus showing the location of the GFP insertions, homeodomain, and CUT, CASP, and transmembrane domains. All GFP sequences used in this study have artificial introns (not shown in the schematics). (*C*) Reporter expression in L4 animals (head, lateral views). (*Left*) *ceh-44(ot1015[ceh-44::GFP])* showing pan-neuronal nuclear expression. (*Right*) *cone-1(syb5500[cone-1::oxGFP])* showing broad puncta. (*D*, *left*) Reporter expression overlap in L4 animals (head, lateral view, posterior bulb pharynx) of *cone-1(syb5500[cone-1::oxGFP])* and *aman-2::tagRFP[pwIs1022]*. (*Right*) Pearson's correlation coefficient measurements for colocalization of CONE-1::GFP with AMAN-2::tagRFP. The overlap of CONE-1 (CASP of nematodes) with AMAN-2 punctae is not perfect, consistent with localization to distinct Golgi subcompartments, where proteins from different compartments may show limited overlap (Norris et al. 2017). (*E*,*F*, *left*) Expression of *cone-1(syb5500[cone-1::oxGFP])* (*E*) and *cone-1(syb6898[cone-1::T2A::GFP::H2B])* (*F*). (*Right*) Merged green/DIC channels showing expression excluded from the nervous system (single Z-slice). Lateral views of the worm head at the L4 stage are shown. The dashed contour outlines the nerve ring region. (*G*) Temporal expression analysis in *cone-1(syb5500[cone-1::oxGFP])* across different embryonic stages: 2 cell, 44 cell, comma, twofold, and threefold. Scale bars, 10 μm.

**Figure 2.**
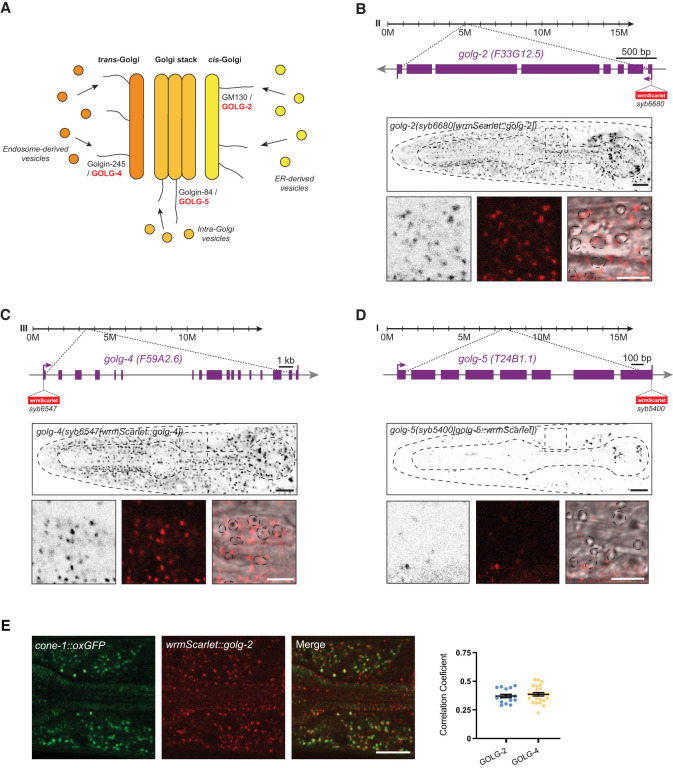
*C. elegans* golgin proteins show ubiquitous and cell type-specific expression patterns. (*A*) Different golgins localize to different Golgi compartments. *C. elegans* orthologs that are tagged in this study are labeled in red. Schematic adapted with permission from Lowe (2019). (*B*–*D*) Schematic representations of the golgin loci showing wrmScarlet insertion locations. Lateral head images of L4 animals showing expression of *golg-2(syb6680[wrmScarlet::golg-2])* (*B*), *golg-4(syb6547[wrmScarlet::golg-4])* (*C*), and *golg-5(syb5400[golg-5::wrmScarlet])* (*D*) (single Z-slices). Magnified images of the area between pharyngeal bulbs show GOLG fluorophore signals in black, red, or red overlaid with DIC channel. Stippled circles in DIC channel images outline cell nuclei. (*E*, *left*) Reporter expression overlap in L4 animals of *cone-1(syb5500[cone-1::oxGFP])* and *golg-2(syb6680[wrmScarlet::golg-2])* (head, lateral view, isthmus). (*Right*) Pearson's correlation coefficient measurements for colocalization of CONE-1::GFP with golgin proteins. (ER) Endoplasmic reticulum. Scale bars, 10 μm; *B–D* zoomed images, 5 μm.

The unusual structure of this locus raises many currently unresolved questions: Which parts of the transcripts are made into proteins? Are those two isoforms really the product of alternative splicing or are they independent transcripts? Are the alternative transcripts generated in an overlapping or mutually exclusive manner? How is this alternative splicing regulated? Why are these two gene products coupled in this manner? Here, we provide answers to several of these questions using the conserved, golgin/homeobox gene locus structure in *C. elegans* as a model (Fig. 1B). We placed the regulation of this locus into the context of control of pan-neuronal gene expression, finding that in *C. elegans* the *ceh-44/CUX* isoform of this locus is produced exclusively in the nervous system via the action of a pan-neuronal-expressed, alternative splicing factor, UNC-75, the *C. elegans* homolog of the vertebrate CELF splicing regulators. This splicing pattern results in the exclusion of the alternative, *cone-1/CASP-*encoding transcript that codes for the *C. elegans* homolog of the CASP golgin from the nervous system. We found that another *C. elegans* golgin protein also displays cell type-specific expression patterns, thereby uncovering the cellular diversity of the composition and, possibly, function of the Golgi apparatus.

## Results and Discussion

### The *cone-1&ceh-44* locus generates two differentially expressed and localized proteins: Golgi-localized CONE-1/CASP and nuclear CUT homeodomain CEH-44/CUX

In *C. elegans*, *ceh-44/CUX* represents the only member of the CUX subfamily of CUT homeobox genes characterized by the presence of multiple CUT domains (Burglin and Cassata 2002). In vertebrates, the orthologous *Cux1* locus produces an alternative transcript that encodes CASP, a single-pass transmembrane protein that localizes to the Golgi apparatus (Fig. 1A; Gillingham et al. 2002). Likewise, by sequence similarity, one can infer that the *C. elegans* locus encoding *ceh-44/CUX* also produces the ortholog of vertebrate CASP, which we named “*cone-1*” (CASP of nematodes). This intertwined gene structure of a golgin and a CUX homeodomain protein can be observed throughout protostome and deuterostome lineages, ranging from nematodes to humans (Supplemental Fig. S1). On an evolutionary timescale, the intertwining of these two loci coincided with the multiplication of CUT domains in an ancestral CUT homeodomain protein that originated at the base of the bilaterians with only a single CUT domain (Burglin and Cassata 2002; Brauchle et al. 2018).

We have previously reported the pan-neuronal expression pattern of *ceh-44/CUX* by tagging *ceh-44/CUX* with a *gfp* reporter via CRISPR/Cas9-mediated genome engineering (Fig. 1B; Leyva-Díaz and Hobert 2022). However, the expression and potential subcellular localization of *cone-1/CASP* have not been examined. To address this question, we CRISPR/Cas9-engineered a translational reporter allele, *cone-1(syb5500)*, by inserting GFP at the C-terminal end of CONE-1/CASP (Fig. 1B). Because, based on its sequence, we predicted CONE-1/CASP to be a transmembrane protein with its C-terminal portion present in the acidic environment of the Golgi apparatus, we used a worm codon-optimized GFP form (oxGFP) engineered to maintain its structure under acidic conditions. We found that CONE-1::oxGFP is broadly expressed throughout the nematode body, displaying a marked punctate pattern, contrasting the nuclear pan-neuronal expression of the CEH-44/CUX isoform of this locus (Fig. 1C). Using an RFP-marked Golgi protein, AMAN-2 (Norris et al. 2017), we found an overlap of CONE-1/CASP and AMAN-2 localization, indicating that, like vertebrate CASP, the nematode CONE-1 protein is a Golgi-resident protein (Fig. 1D).

CONE-1/CASP puncta are conspicuously absent from neuronal cells (Fig. 1E) and hence display a mutually exclusive expression with *ceh-44/CUX*. To validate this observation, we generated a *cone-1/CASP* reporter allele, *cone-1(syb6998)*, in which we inserted a nuclear-localized GFP reporter cassette at the 3′ end of the *cone-1/CASP* transcript, separated from CONE-1/CASP protein by a “ribosomal skip” T2A peptide (Ahier and Jarriault 2014). This nuclear-directed reporter allows for independent identification of sites of expression and validates exclusion of CONE-1/CASP expression from the nervous system (Fig. 1F).

Intriguingly, we found a notable switch in CEH-44/CUX and CONE-1/CASP expression during embryonic development. As we have described previously, CEH-44::GFP expression initiates promptly after neuronal birth at the embryonic comma stage (Leyva-Díaz and Hobert 2022). In contrast, we detected CONE-1/CASP expression, as assessed with the translational CONE-1::oxGFP reporter, as early as the 2 cell stage, with a broad, seemingly ubiquitous expression pattern across all proliferative embryonic stages (Fig. 1G), followed by exclusion of CONE-1 and onset of CEH-44 expression in the maturing, postmitotic nervous system.

### Cell type specificity of golgin expression

The neuron-excluded expression of the CONE-1/CASP protein prompted us to test whether other *C. elegans* golgin proteins may also display cell type-specific expression profiles. To this end, we considered *C. elegans* orthologs of three different golgin proteins described in other systems (GM130, golgin-245, and golgin-84), each labeling distinct Golgi compartments (GM130 *cis*-Golgi, golgin-84 intra-Golgi vesicles, and golgin-245 *trans*-Golgi) (Fig. 2A; Munro 2011; Lowe 2019). We termed these orthologs *golg-2* (GM130 ortholog), *golg-4* (golgin-245), and *golg-5* (golgin-84). wrmScarlet, a worm codon-optimized version of mScarlet (El Mouridi et al. 2017), was inserted either right after the initiation codon of the gene (*golg-2* and *golg-4*) or right before the termination codon of the gene (*golg-5*) using CRISPR/Cas9 genome engineering (Fig. 2B–D). Punctate expression patterns were observed in each of the three strains. In all three cases, expression persists throughout all developmental stages into adulthood. In nonneuronal cells, these puncta overlapped with CONE-1/CASP localization, corroborating the Golgi localization of these proteins (Fig. 2E). Intriguingly, although GOLG-2 and GOLG-4 are broadly, if not ubiquitously, expressed in the adult animal, GOLG-5 protein appears to be expressed only in a subset of cell types throughout the animal (Fig. 2D). Therefore, we have uncovered here the first two examples of cell type-specific golgin protein expression: GOLG-5 and CONE-1.

To assess CONE-1/CASP protein function, we generated a *cone-1/CASP*-specific mutant allele through the insertion of a premature stop codon in the first *cone-1/CASP-*specific exon (Supplemental Fig. S2A). No obvious developmental or behavioral defects were observed in animals carrying this *cone-1/CASP* mutant allele. Moreover, we found that neither the expression of *ceh-44/CUX* nor its function—as assessed by the expression of *ric-19*/*ICA1*, a pan-neuronal gene controlled by *ceh-44/CUX* (Leyva-Díaz and Hobert 2022)—is altered in animals lacking *cone-1/CASP* (Supplemental Fig. S2B,C). The absence of the *cone-1/CASP* phenotype aligns with the lack of phenotypes observed after knockout of the yeast homolog of CONE-1/CASP, called COY, or other golgins in mice or flies (Matsukuma et al. 1999; McGee et al. 2017; Park et al. 2022). Similarly, animals carrying deletion or nonsense alleles of *golg-2*, *golg-4*, and *golg-5* generated by the *C. elegans* knockout consortia (Au et al. 2019) appear viable, consistent with viable phenotypes of fly and mouse homologs of these genes.

### Defining CONE-1- and CEH-44-coding regions

**Figure 3.**
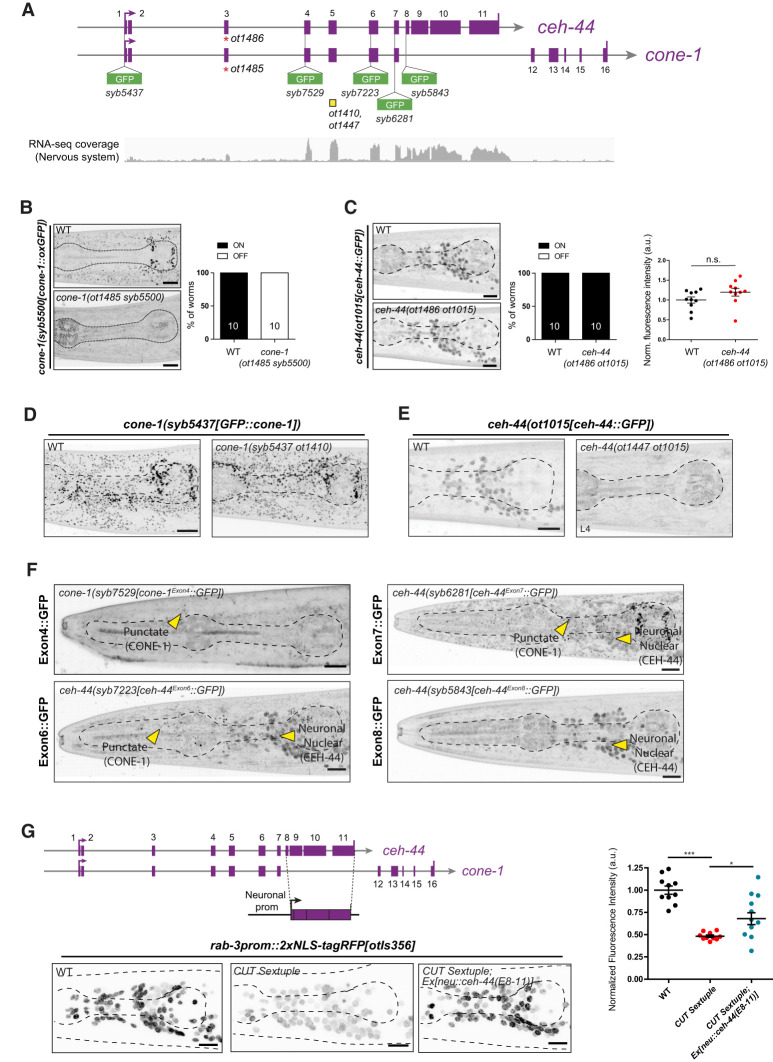
Mapping coding sequence of *cone-1* and *ceh-44* via insertion of GFP into distinct exons. (*A*) Schematic representation of the *cone-1&ceh-44* gene locus showing GFP insertion locations and mutant alleles (yellow boxes indicate deletions, and red asterisks indicate early stop codons). RNA-seq coverage track from isolated nervous system tissue is shown at the *bottom* (RNA-seq, average, *n* = 3). (*B*,*C*) *cone-1(syb5500[cone-1::oxGFP])* (*B*) and *ceh-44(ot1015[ceh-44::GFP])* (*C*) reporter expression in L4 animals (head, lateral views) in wild type (*top*) and mutants carrying an early stop codon in exon 3 (*bottom*). Note that the *ot1485* (*B*) and *ot1486* (*C*) mutant alleles are designed to maintain frame and harbor the same molecular lesion. The percentages of animals that express the reporter (on/off) are indicated. CRISPR allele fluorescence intensity in head neurons is quantified in *C*. The data are presented as individual values, with each dot representing the expression level of one worm with the mean ± SEM indicated. Unpaired *t*-test. *n* = 10 for all genotypes. (*D*,*E*) *cone-1(syb5437[GFP::cone-1])* (*D*) and *ceh-44(ot1015[ceh-44::GFP])* (*E*) reporter expression in L4 animals (head, lateral views) in wild type (*left*) and mutants carrying an exon 5 deletion (*right*). Note that the *ot1410* (*D*) and *ot1447* (*E*) mutant alleles are designed to maintain frame and harbor the same molecular lesion. See Supplemental Figure S3C for sequence details. (*F*) Reporter expression in L4 animals of *cone-1(syb7529[cone-1^Exon4^::GFP])* (*top left*), *ceh-44(syb7223[ceh-44^Exon6^::GFP])* (*bottom left*), *ceh-44(syb6281[ceh-44^Exon7^::GFP])* (*top right*), *and ceh-44(syb5843[ceh-44^Exon8^::GFP])* (*bottom right*) (head, lateral views). Yellow arrowheads point to CONE-1/CASP puncta and CEH-44/CUX neuronal nuclear expression. (*G*, *left*) Expression of *rab-3prom1::2xNLS-tagRFP[otIs356]* in wild type (*bottom left*), CUT sextuple mutants (*bottom middle*), and CUT sextuple mutant rescues {pan-neuronal expression of *ceh-44/CUX*-specific exons 8–11, *neu::ceh-44(E8-11)[otEx7645]*; “neu” = *ceh-48* promoter} (*bottom right*). Images are lateral head views of L4 animals (maximum Z-projections). (*Right*) Quantification of fluorescence intensity in head neurons. Each dot represents the expression level within one worm with the mean ± SEM indicated. (Black dots) Wild-type data, (red dots) CUT sextuple mutant data, (blue dots) rescue data. One-way ANOVA followed by Tukey's multiple comparisons test. (*) *P* < 0.05, (***) *P* < 0.001. *n* ≥ 10 for all genotypes. (ns) Not significant, (au) arbitrary units. Scale bars, 10 μm.

Although *ceh-44/CUX* and *cone-1/CASP* probably serve different functions, they are predicted to share a large portion of their N-terminal region. However, the key CEH-44/CUX functional domains (the CUT and homeodomain DNA binding domains) are exclusively encoded within the *ceh-44/CUX*-specific exons. Similarly, vertebrate *Cux1* and *CASP* also share several exons, and the CUT and homeodomain sequences are also encoded by *Cux1*-specific exons (Fig. 1A). To experimentally confirm predictions of gene structures and their cell type-specific expression, we first examined transcripts from the *cone-1&ceh-44* locus in available neuronal RNA-seq data (Leyva-Díaz and Hobert 2022). Confirming our reporter-based expression data, *ceh-44/CUX*-specific exons are well covered by neuronal RNA-seq reads, whereas *cone-1/CASP*-specific exons are absent in neuronal transcripts of this locus (Fig. 3A). Intriguingly, coverage within the shared exons starts only in exon 4, indicating a potential alternative *ceh-44/CUX* start site (Fig. 3A). To test this notion, we introduced an early stop codon in exon 3 and found CEH-44::GFP protein localization and expression levels to be unaffected, whereas CONE-1::GFP protein expression was completely eliminated (Fig. 3A–C). Consistent with the production of a shorter CEH-44/CUX protein lacking exons 1–3, insertion of GFP via CRISPR/Cas9 into the first exon, right after the start codon (Fig. 3A, *syb5437* allele), produced Golgi-localized GFP::CONE-1 expression but no neuronal, nuclear GFP::CEH-44 expression, further corroborating the use of an alternative start site for *ceh-44/CUX* (Fig. 3D, left).

To dissect the structure of CEH-44/CUX and CONE-1/CASP proteins further, we inserted *gfp* at different positions within the *cone-1&ceh-44* endogenous locus (Fig. 3A; Supplemental Fig. S3A). First, CRISPR/Cas9-engineered *gfp* insertion in the *ceh-44/CUX*-specific exon 8, the first exon of *ceh-44/CUX*-specific exons, revealed pan-neuronal and nuclear CEH-44::GFP protein, as expected (Fig. 3F). *gfp* insertions within exon 6 or exon 7, which are shared between *cone-1/CASP* and *ceh-44/CUX*, resulted in simultaneous detection of CONE-1::GFP in nonneuronal cells and CEH-44::GFP in neurons (Fig. 3F). In contrast, only Golgi-localized CONE-1::GFP was observed when *gfp* was engineered into exon 4 (Fig. 3F).

**Figure 4.**
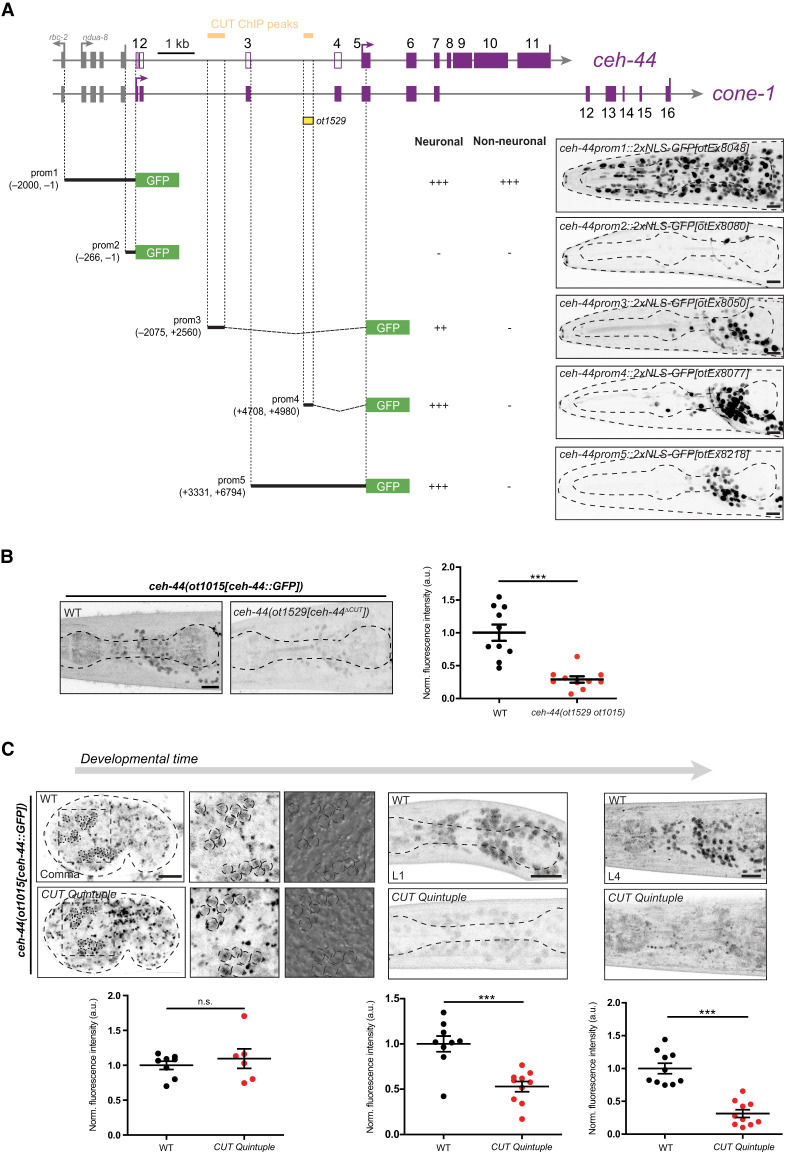
CUT homeobox genes maintain *ceh-44/CUX* expression. (*A*) Schematic representation of *cone-1&ceh-44* locus *cis*-regulatory analysis. CEH-48 and CEH-38 peaks found in the ChIP-seq data sets are displayed in yellow. Promoter 1–5 designs are displayed with coordinates, and neuronal and nonneuronal expression is described as either no expression (−), broad expression (++), or pan-neuronal/ubiquitous expression (+++). (*B*) *ceh-44(ot1015[ceh-44::GFP])* reporter expression in wild-type (*left*) and mutant animals carrying a mutation in the CUT ChIP peak within intron 3 (*ot1529* allele; *right*). (*C*) *ceh-44(ot1015[ceh-44::GFP])* reporter expression at the comma embryonic stage (*left*), larval L1 stage (*middle*), and larval L4 stage (*right*) in wild type (*top*) and CUT quintuple mutants (*bottom*). High-magnification images of comma stage embryos are shown, with dashed circles indicating the location of neuronal *ceh-44* expression. At the *right*, high-magnification Nomarski micrographs are provided, highlighting the presence of nuclear speckles within these cells. (*B*,*C*) Quantification of CRISPR-engineered reporter allele fluorescence intensity in head neurons is shown. The data are presented as individual values, with each dot representing the expression level of one worm with the mean ± SEM indicated. (Black dots) Wild-type data, (red dots) mutant data. Unpaired *t*-test, (***) *P* < 0.001. *n* ≥ 6 for all genotypes. Images show lateral views in all panels (L4 stage in *A*,*B*). (ns) Not significant, (au) arbitrary units. Scale bars, 10 μm.

Although exon 4 is covered in neuronal transcriptomic data (Fig. 3A), there is no potential start codon within exon 4, yet there are three start codons in exon 5 just prior to the GFP insertion within exon 6 (Supplemental Fig. S3B). If any of these start codons indeed represent the beginning of the mature CEH-44, a notion supported by our GFP insertion data, the CEH-44 protein would only entail a small part of the large α-helical structure that characterizes CONE-1 and its yeast and vertebrate CASP homologs (Supplemental Fig. S3A–C). To assess the importance of exon 5 and its embedded start codons, we engineered an in-frame deletion of most codons within this exon, including all potential start codons, using the CRISPR/Cas9 system (Fig. 3A; Supplemental Fig. S3C). We found that in these animals, CEH-44::GFP expression is completely abolished, whereas CONE-1::GFP expression remained unaffected (Fig. 3D,E). Taken together, our data support an updated *cone-1&ceh-44* locus structure with different start sites for each gene (Fig. 4A): The *cone-1/CASP* initiation codon is present in exon 1, while the *ceh-44/CUX* initiation codon resides within exon 5. As we describe in the next section, the initiation of the shorter *ceh-44/CUX* transcript may be an autoregulatory effect.

Finally, we sought to investigate the requirement of shared exons for *ceh-44/CUX* function. We have shown previously that the expression of pan-neuronal genes, such as the small GTPase *rab-3*, is significantly reduced in animals lacking all the neuronal CUT genes, including *ceh-44/CUX* (CUT sextuple mutants). Overexpression of a *ceh-44/CUX* construct containing exons 1–11 rescued pan-neuronal gene expression (Leyva-Díaz and Hobert 2022). Here, we found that a rescuing construct containing only *ceh-44/CUX*-specific exons (exons 8–11) also rescues *rab-3* expression (Fig. 3G), suggesting that the exons shared by *cone-1* and *ceh-44* might not be required for *ceh-44/CUX* function.

### Different regulatory elements control the *ceh-44/CUX* pan-neuronal expression pattern

After defining the complex structure and expression of the *cone-1&ceh-44* locus, we sought to deconstruct its *cis-*regulatory architecture. To this end, we constructed reporter gene fusions that interrogate the *cis-*regulatory content within both upstream and intronic regions of the *cone-1&ceh-44* locus. First, we generated a reporter construct that contains 2 kb upstream of the primary *cone-1&ceh-44* transcript (*ceh-44prom1*). We found this reporter to drive expression broadly in both neuronal and nonneuronal cells (Fig. 4A). This broad expression pattern is consistent with the *cone-1&ceh-44* locus being the second cistron in an operon (Blumenthal et al. 2002), with *ndua-8* upstream, suggesting that the promoter driving this expression may be operon-derived. To corroborate that this transcript is indeed produced in all cells, we engineered an endogenous reporter for this upstream region by inserting a *gfp::H2B::SL2* cassette just before the *cone-1/CASP* initiation codon (Supplemental Fig. S4A). Due to the *trans*-splicing driven by the bicistronic SL2 linker (Spieth et al. 1993; Huang et al. 2001), this reporter served as a GFP transcriptional reporter for the *cone-1&ceh-44* upstream promoter. Expression analysis across different embryonic and larval stages unveiled widespread, ubiquitous expression in both neuronal and nonneuronal cells, commencing at the 2 cell embryo stage, possibly due to maternal deposition, and persisting through adulthood (Supplemental Fig. S4B). These observations suggest that this upstream region may function as an initiation and maintenance regulatory element for the locus.

Interestingly, the *cone-1&ceh-44* locus contains within its second and third intron predicted CUT homeodomain binding sites (DNA-binding sites of CUX and ONECUT proteins) (Jolma et al. 2013) that are typically present in pan-neuronal genes (Leyva-Díaz and Hobert 2022). Indeed, chromatin immunoprecipitation (ChIP) analysis conducted by the modENCODE Consortium (Davis et al. 2018) demonstrated binding of the CUT factors CEH-48 and CEH-38 to these two different intronic regions. We found that transgenic reporters containing either of these binding regions (*ceh-44prom3* and *ceh-44prom4*) drive robust neuronal expression (Fig. 4A). Using CRISPR/Cas9 technology, we deleted the CUT ChIP peak within intron 3 in the *ceh-44/CUX* GFP-tagged endogenous locus, resulting in a significant reduction in *ceh-44/CUX* expression (Fig. 4B). Such reduction of *ceh-44::gfp* expression is also observed upon genetic removal of the five neuronally expressed ONECUT genes (*ceh-38*, *ceh-48*, *ceh-39*, *ceh-21*, and *ceh-41*), which together with *ceh-44/CUX* redundantly control pan-neuronal gene expression (Fig. 4C; Leyva-Díaz and Hobert 2022).

We next asked whether the CUT regulation of the *ceh-44/CUX* locus may be a reflection of CUT genes maintaining proper CEH-44::GFP expression after the initial onset of *ceh-44/CUX* expression. Indeed, we found that although expression of *ceh-44/CUX* is significantly reduced throughout all larval stages in quintuple CUT mutant animals, expression remained unaffected at the embryonic comma stage during the onset of *ceh-44/CUX* expression (Fig. 4C). Therefore, we conclude that the CUT genes are required for the maintenance of *ceh-44/CUX* expression through binding to the intronic elements present in the *cone-1&ceh-44* locus. We surmise that this autoregulation is what produces the shorter *ceh-44/CUX* isoform that is observed in RNA-seq data in the nervous system (Fig. 3A) and that we mapped via GFP insertions into the locus (Fig. 3F).

### UNC-75/CELF is required for pan-neuronal *ceh-44/CUX* transcript production

**Figure 5.**
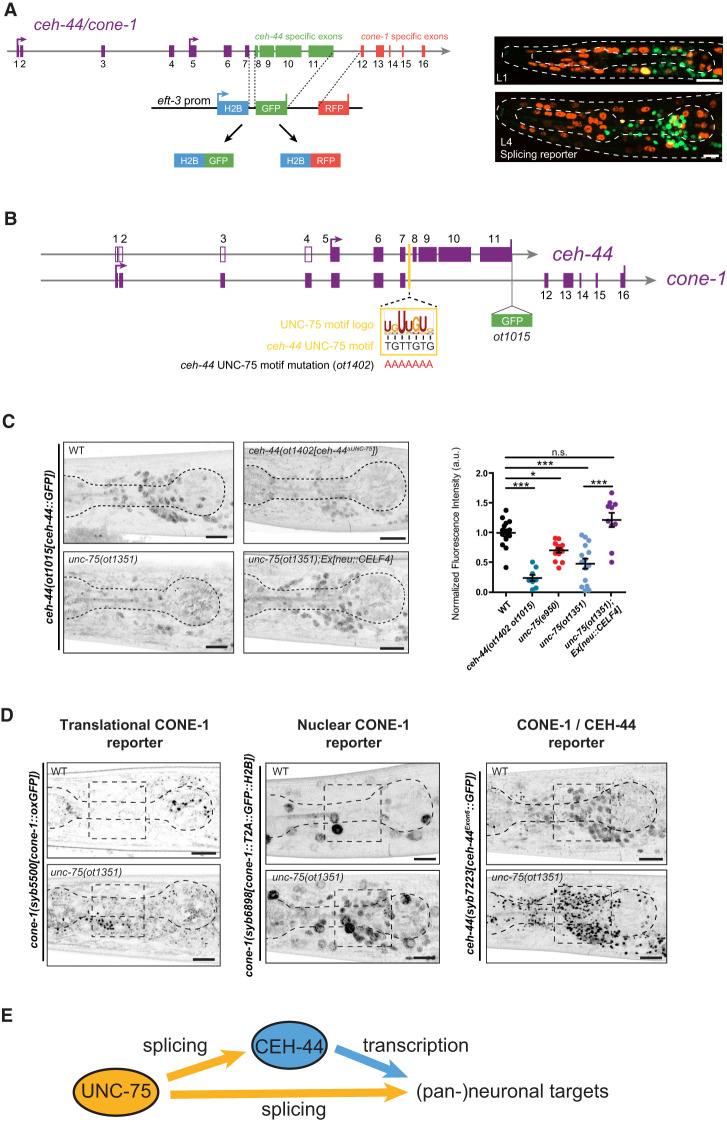
The pan-neuronal CELF homolog *unc-75/CELF* is required for *ceh-44/CUX* production. (*A*, *left*) Schematic representation of the splicing reporter cassette. GFP or RFP are localized to the nucleus via H2B and capture the alternative splicing activity of the gene locus. (*Right*) Images showing lateral head views at the L1 (*top*) and L4 (*bottom*) larval stages (maximum Z-projections). Although rare, instances of both reporters coexpressing in the same cell are observed, typically as a single yellow cell per worm. These occurrences are inconsistent in terms of location, often appearing in nonneuronal cells, and vary between individuals. Despite this, the general splicing pattern remains reproducible and invariant from one animal to the next. (*B*) Schematic representation of *cone-1&ceh-44* gene locus showing GFP insertion, the UNC-75/CELF motif logo, location within intron 7, and motif mutation in *ceh-44(ot1402 ot1015)*. (*C*) *ceh-44(ot1015[ceh-44::GFP])* reporter expression in wild type (*top left*), *ceh-44(ot1402 ot1015)* (*top middle*), *unc-75(ot1351)* (*bottom left*), and *unc-75(ot1351); neu::CELF4[otEx8142]* (*bottom middle*). (*Right*) Quantification of CRISPR allele fluorescence intensity in head neurons. Each dot represents the expression level within one worm with the mean and ± SEM indicated. (Black dots) Wild-type data, (teal dots) *ceh-44(ot1402 ot1015)* data, (red dots) *unc-75*(*e950*) data, (blue dots) *unc-75(ot1351)* data, (purple dots) *unc-75(ot1351); neu::CELF4[otEx8142]* data. One-way ANOVA followed by Tukey's multiple comparisons test. (*) *P* < 0.05, (***) *P* < 0.001. *n* ≥ 10 for all genotypes. (*D*) *cone-1(syb5500[cone-1::oxGFP])* (*left*), *cone-1(syb6898[cone-1::T2A::GFP::H2B])* (*middle*), and *ceh-44(syb7223[ceh-44Exon6::GFP])* (*right*) reporter allele expression in wild-type (*top*) and *unc-75(ot1351)* mutant (*bottom*) animals (head, lateral view, L4 stage). The dashed square outlines the nerve ring region. (*E*) Summary illustrating the feed-forward architecture to control the production of pan-neuronal targets through UNC-75/CELF splicing and CEH-44/CUX transcriptional regulation. Supplemental Figure S6 shows a more expansive summary of the findings of this work. (ns) Not significant, (au) arbitrary units. Scale bars, 10 μm.

Apart from gaining insights into the transcriptional regulation of the *cone-1&ceh-44* locus, we sought to define the mechanisms underlying the alternative splicing of *ceh-44/CUX* and *cone-1/CASP.* We initially focused this analysis on the two introns that precede the first isoform-specific exons of *ceh-44/CUX* and *cone-1/CASP*, respectively (Fig. 5A), asking whether these introns may contain splicing regulatory sequences. To this end, we developed a two color splicing reporter cassette that provides a fluorescent readout of splicing regulation. The reporter cassette is expressed under control of a heterologous ubiquitous promoter and places GFP and RFP behind each of these introns, in lieu of the first isoform-specific exons of *ceh-44/CUX* and *cone-1/CASP*, respectively (Fig. 5A). Hence, tissue-specific alternative splicing should become evident through tissue-specific expression of GFP and RFP. Mirroring the expression pattern of the endogenous proteins, we found that GFP expression from this reporter cassette is indeed detected in all neuronal cells, whereas RFP is detected in nonneuronal cell types (Fig. 5A).

Because *cone-1/CASP* expression is ubiquitous at early embryonic stages and *ceh-44/CUX* expression becomes produced upon postmitotic neuronal differentiation, we hypothesized that a pan-neuronal splicing factor might be involved in the generation of neuron-specific *ceh-44/CUX* and the active exclusion of *cone-1/CASP*. The *C. elegans* homolog of the CELF family of RRM domain-containing splicing factors, UNC-75/CELF, constitutes a pan-neuronally expressed splicing factor (Loria et al. 2003; Kuroyanagi et al. 2013a,b; Norris et al. 2014; Chen et al. 2016; Koterniak et al. 2020). Because pan-neuronal expression was only inferred from a transgenic reporter construct (Loria et al. 2003), we sought to confirm this expression pattern with a CRISPR/Cas9-engineered endogenous reporter allele. We observed expression of this reporter allele in all neurons, starting at the embryonic comma stage, coinciding with the onset of *ceh-44/CUX* expression (Supplemental Fig. S5A). *unc-75/CELF* pan-neuronal expression is maintained through all larval stages and into adulthood, aligning with the expression pattern of *ceh-44/CUX* and positioning *unc-75/CELF* as a strong candidate to regulate the *cone-1&ceh-44* splicing event. Supporting this notion, animals carrying the canonical *unc-75(e950)* mutant allele, a deletion affecting two of the three UNC-75/CELF RNA binding RRM motifs (Supplemental Fig. S5A; Loria et al. 2003), show a strong reduction in *ceh-44/CUX* expression (Fig. 5C). We also engineered a more unambiguous null allele, *unc-75(ot1351)*, in which all three RRMs are removed (Supplemental Fig. S5A). *unc-75(ot1351)* animals display locomotory defects similar to those observed in *unc-75(e950)* animals (Supplemental Fig. S5B), and expression of *gfp-*tagged *ceh-44/CUX* was also significantly reduced (Fig. 5C).

UNC-75/CELF regulation of *ceh-44/CUX* splicing is likely direct. We identified a predicted UNC-75/CELF binding motif (Koterniak et al. 2020) within intron 7 of the *cone-1&ceh-44* locus, between the shared exons and *ceh-44/CUX*-specific exons (Fig. 5B). Furthermore, an UNC-75/CELF binding peak was observed in this intron through CLIP-seq analysis (J. Calarco, pers. comm.). We assessed the functional relevance of this UNC-75/CELF binding site through deletion from the endogenous *cone-1&ceh-44* locus (Fig. 5B, *ot1402* allele). Mutation of the UNC-75/CELF binding site significantly reduced *ceh-44/CUX* expression, as visualized with the endogenous *ceh-44::gfp* reporter allele (Fig. 5C).

Consistent with this loss of *ceh-44/CUX* expression, transcription of the pan-neuronal gene *ric-19/ICA1* is significantly reduced in *unc-75/CELF* mutants, indicating defective *ceh-44/CUX* function (Supplemental Fig. S5C). Expression of a full-length *ceh-44/CUX*-rescuing construct (exons 5–11) rescues *ric-19* expression (Supplemental Fig. S5C), confirming our model in which UNC-75 controls *ceh-44* production, which in turn regulates pan-neuronal gene expression. Synaptic transmission defects in *unc-75/CELF* mutants can be rescued by expressing a human *CELF4* cDNA under the control of the pan-neuronal *unc-75/CELF* promoter (Loria et al. 2003). Here we demonstrate that the same construct can rescue the *ceh-44/CUX* expression phenotype of *unc-75/CELF* mutant animals (Fig. 5C), confirming the conservation of UNC-75/CELF function across phylogeny.

Given these findings, we reasoned that if UNC-75/CELF indeed controls a neuron-specific splicing event that promotes *ceh-44/CUX* transcript production at the expense of *cone-1/CASP*, then *unc-75/CELF* mutant animals should exhibit ectopic *cone-1/CASP* expression in the nervous system. We observed such ectopic expression using three different *cone-1/CASP* reporter alleles (Fig. 5D). Hence, UNC-75/CELF promotes *ceh-44/CUX* transcript production in the nervous system while excluding the production of an alternative, *cone-1/CASP-*encoding transcript.

### Conclusions

In this study, we have deconstructed the architecture and regulation of a phylogenetically deeply conserved and highly unusual genetic locus that produces two distinct proteins with no apparent functional commonalities. We propose the following model for the regulation of transcript production from the *cone-1&ceh-44* locus (Supplemental Fig. S6): Primary transcript production from this locus is directed to all cells by factors controlling the upstream promoter, which is active throughout the life of the organism. This transcript is spliced via as yet unknown means to produce only the mature *cone-1* transcript. Upon neuronal differentiation and the onset of UNC-75/CELF expression, a splicing switch mediated by UNC-75/CELF results in *ceh-44/CUX* production in neurons, at the expense of *cone-1/CASP*. In neurons, *ceh-44/CUX* expression is then maintained by CUT factors, including CEH-44/CUX, binding to intronic *cis*-regulatory elements upstream of the *ceh-44/CUX* transcript start. Thus, sustained pan-neuronal expression levels are primarily controlled by an intronic element that promotes expression in neurons, whereas alternative splicing by UNC-75/CELF further refines this structure of the transcript (Supplemental Fig. S6).

UNC-75/CELF, a conserved RNA-binding protein, plays a crucial role in the regulation of RNA metabolism, including the regulation of splicing (Dasgupta and Ladd 2012; Kuroyanagi et al. 2013a,b; Norris et al. 2014; Chen et al. 2016; Koterniak et al. 2020). In *C. elegans*, UNC-75, along with other splicing factors, orchestrates splicing networks that contribute to neuronal diversity (Norris et al. 2014; Chen et al. 2016; Koterniak et al. 2020). Interestingly, DNA elements proximal to almost half (235) of the 534 genes whose primary mRNA transcripts are bound by UNC-75 in CLIP-seq analysis (Chen et al. 2016) show CHIP-seq binding by several CUT homeodomain proteins (Davis et al. 2018). Hence, through directing CEH-44/CUX CUT homeodomain expression to the nervous system through alternative splicing, UNC-75/CELF orchestrates in a feed-forward manner the production of CEH-44/CUX-dependent primary transcripts that then serve as substrates for splicing (Fig. 5E).

We found that *cone-1/CASP* is actively excluded from the nervous system and also identified another golgin with a cell type-specific expression pattern. This contrasts the supposed ubiquitous expression of other golgins, which are generally thought to capture vesicles to promote their transport along the Golgi apparatus (Munro 2011; Gillingham and Munro 2016; Lowe 2019). The identification of two Golgi proteins with cell type-specific expression patterns points to cell type-specific cargo transport along the Golgi apparatus.

Notably, the *cone-1&ceh-44* locus structure is highly conserved throughout evolution. However, the regulatory mechanisms governing this locus in vertebrates remain unknown, including whether CELF factors play a similar role. Both vertebrate CASP and *Cux1* are expressed in vertebrate neuronal and nonneuronal cells (Lievens et al. 1997; Rong Zeng et al. 2000; Weiss and Nieto 2019; Suzuki et al. 2022), but whether or to what extent expression of the two isoforms overlaps or is mutually exclusive has not been determined with single-cell resolution. Our finding of a strictly mutually exclusive expression of CONE-1 and CEH-44 in *C. elegans* allows the important conclusion that neither protein can be involved in the function of the other protein, a speculation fueled by an initially reported in vitro interaction of CASP and Cux1 (Lievens et al. 1997). The sequences shared between the two proteins can also not be involved in controlling the localization of either protein because both proteins are located at distinct subcellular sites. At this point, we do not know whether the shared sequences carry any functional weight. Although we found the shared sequences not to be important for the presently known functions of CEH-44, there may be other unknown functions of this protein that require these sequences.

If these shared sequences are not important for protein function, why is this unusual locus structure so conserved? Our best current explanation for such preservation is that the *cis*-regulatory architecture of the locus, established after the translocation that fused the two loci early in metazoan evolution, has locked the two loci into a configuration that cannot be separated without losing the proper expression and hence function of either isoform. Consistent with this notion, multiple cases of blocks of deeply conserved microsynteny have been discovered in animal genomes, and it has been proposed that such constellations are conserved because transcriptional enhancers controlling developmental genes (in this case, the CUT sites in introns 2 and 3 that promote *ceh-44* expression) are contained within nearby bystander genes (*cone-1*) (Irimia et al. 2012), thereby preventing separation of the loci over evolutionary timescales. Studies on the *cis*- and *trans*-regulatory apparatus of the orthologous CASP/CUX in vertebrates may further corroborate this hypothesis.

## Materials and methods

### *C. elegans* strains and handling

Worms were grown at 20°C on nematode growth medium (NGM) plates seeded with *Escherichia coli* (OP50) bacteria as a food source (Brenner 1974). The wild-type strain used was Bristol variety, strain N2. A complete list of strains used in this study is in Supplemental Table S1. Information about transgenes and alleles generated via CRISPR/Cas9-based genome engineering is detailed in the Supplemental Material.

### Automated worm tracking

For the tracking of *unc-75/CELF* mutants and controls, tracking was performed using the WormLab automated multiworm tracking system (MBF Bioscience) (Roussel et al. 2014) at room temperature. In each plate, five young adult animals were recorded for 5 min and tracked on NGM plates with no food. Videos were segmented to extract the worm contour and skeleton for phenotypic analysis. Raw WormLab data were exported to Prism (GraphPad) for further statistical analysis. Statistical significance between each group was calculated using one-way ANOVA followed by Tukey's multiple comparisons test.

### Microscopy

Worms were anesthetized using 100 mM sodium azide and mounted on 5% agarose on glass slides. Images were acquired using a Zeiss confocal microscope (LSM 980) and a Zeiss fluorescence microscope (Axio Imager.Z1). Image reconstructions were performed using Zeiss Zen software tools. Maximum intensity projections of representative images are shown. Fluorescence intensity was quantified using ImageJ software (Schneider et al. 2012). Figures were prepared using Adobe Illustrator.

### Colocalization analysis

Colocalization analysis were performed by preparing the fluorescence microscopy images with ImageJ software (Schneider et al. 2012) and analyzing them with the JACoP plug-in. Their relative degree of colocalization was expressed in Pearson's correlation coefficient (Adler and Parmryd 2010).

### Visualization of averaged RNA-seq coverage tracks

Alignment files from our previous nervous system transcriptional profiling (Leyva-Díaz and Hobert 2022) were used to generate an averaged coverage track with bedtools and then visualized with the Integrative Genomics Viewer (IGV) genome browser (Robinson et al. 2011).

### Quantification and statistical analysis

All microscopy fluorescence quantifications were done in ImageJ software (Schneider et al. 2012). For all images used for fluorescence intensity quantification, the acquisition parameters were maintained constant among all samples (same pixel size and laser intensity), with control and experimental conditions imaged in the same imaging session. For quantification of head neurons, fluorescence intensity was measured in maximum intensity projections using a single rectangular region of interest from anterior to posterior bulb. A standard threshold was assigned to all the control and experimental conditions being compared. All statistical tests for fluorescence quantifications and behavior assays were conducted using Prism (GraphPad) as described in the figure legends.

## Supplemental Material

Supplement 1
