# Supplementary material for: Alternative splicing controls pan-neuronal homeobox gene expression
Source: Genes Dev. 2025 Feb 1;39(3-4):209–20. doi: 10.1101/gad.352184.124 (PMC11789633; doi:10.1101/gad.352184.124)
Supplement: Supplement 1 [file Supplemental_Material.docx]

**SUPPLEMENTAL MATERIAL**

**Supplemental Materials and Methods** includes detailed information about transgenes and CRISPR allele generation.

**Supplemental Table S1** includes all the *C. elegans* strains used in this study.

**Supplemental Figure S1** related to Figure 1, shows the evolutionary history of the CASP/CUX locus.

**Supplemental Figure S2** related to Figure 2, shows the characterization of *cone-1/CASP* mutants.

**Supplemental Figure S3** related to Figure 3, shows CEH-44/CUX protein structure and sequence details.

**Supplemental Figure S4** related to Figure 4, shows the expression analysis of an endogenous *cone-1/CASP* transcriptional reporter.

**Supplemental Figure S5** related to Figure 5, shows the characterization of new *unc-75/CELF* reporter and null alleles.

**Supplemental Figure S6** related to Figure 5, shows a more extensive schematic of the findings of this work.

**Supplemental Table 1** provides a list of strains used in this study.

**SUPPLEMENTAL MATERIALS AND METHODS**

**CRISPR/Cas9-based genome engineering**

*cone-1(ot1485 syb5500), ceh-44(ot1486 ot1015)*, *ceh-44(ot1529 ot1015), unc-75(ot1351 ot1015), cone-1(syb5437 ot1410), cone-1(ot1282), cone-1(ot1287), ceh-44(ot1447 ot1015), ceh-44(ot1402 ot1015), cone-1(ot1502)* were generated using Cas9 protein, tracrRNA, and crRNAs from IDT, as previously described (Dokshin et al. 2018). For *cone-1(ot1485 syb5500)* and *ceh-44(ot1486 ot1015)*, one crRNA (agtttgacggtcttctagca) and a ssODN donor (tgatcaaggcatttcaaagcgagtttgacgggtctttaagcacggagcacagctgctgaaaatgcactgattga) were used to introduce a nonsense mutation in *cone-1&ceh-44* exon 3 (+3262). For c*eh-44(ot1529 ot1015),* two crRNAs (agcagtattggcgggacgcg and ctaatacggacgggtgacgg) and a ssODN (cttctcatgggggttgttggtgcctcccgccccgccggcggctcttttttgtgctcaatttttcttgatc) were used to delete 224 bp (+4728, +4952) overlapping with the CEH-38/48 ChIP peak on *ceh-44*. For *unc-75(ot1351 ot1015)* two crRNAs (ttaaaacggttcgaagttgg and taatcaatcaataatggcac) and a ssODN as a donor (ttaatggcctaacattttgtatttctaggccaccaccattattgattgattatatatgtatttttgtatt) were used to generate a 7665 bp (+1497, +9162) deletion in *unc-75*. For *cone-1(syb5437 ot1410)* and *ceh-44(ot1447 ot1015)*, two crRNAs (ctttttttcgacttcgttga and gctaaacacaaaattgtatg) and a ssODN (caagattttcaatttttcagatcgaaaacgccgtcaagcataagattgtatgcggtttactgagagaatttacatattcccga) were used to delete *cone-1&ceh-44* exon 5 (+6583, +6817). For *cone-1(ot1282)* and *cone-1(ot1287)* a crRNA (ataaatttcaggacgaaatg) and a ssODN (tttttcgtcaaaaaaggttatcataaatttcaggatgatgaggagcaagaagccgaattgacagtgctcaaag) were used to introduce a STOP and a frameshift in *cone-1* exon 12 (at +13136). For UNC-75 binding site mutation in *ceh-44(ot1402 ot1015)*, the UNC-75 motif UGUUGUG (+8992, +8998) was mutated to AAAAAAA. For *cone-1(ot1502[GFP::H2B::SL2::cone-1]),* a crRNA (ctcttgagacgatttccata) and a ssODN (ttaaataattcgattaatttcttatttcagaccttatgagtaaaggagaagaacttttcactggagttgtcccaattcttgttgaattagatggtgatgttaatgggcacaaattttctgtcagtggagagggtgaaggtgatgcaacatacggaaaacttacccttaaatttatttgcactactggaaaactacctgttccatgggtaagtttaaacatatatatactaactaaccctgattatttaaattttcagccaacacttgtcactactttctgttatggtgttcaatgcttctcgagatacccagatcatatgaaacggcatgactttttcaagagtgccatgcccgaaggttatgtacaggaaagaactatatttttcaaagatgacgggaactacaagacacgtaagtttaaacagttcggtactaactaaccatacatatttaaattttcaggtgctgaagtcaagtttgaaggtgatacccttgttaatagaatcgagttaaaaggtattgattttaaagaagatggaaacattcttggacacaaattggaatacaactataactcacacaatgtatacatcatggcagacaaacaaaagaatggaatcaaagttgtaagtttaaacatgattttactaactaactaatctgatttaaattttcagaacttcaaaattagacacaacattgaagatggaagcgttcaactagcagaccattatcaacaaaatactccaattggcgatggccctgtccttttaccagacaaccattacctgtccacacaatctgccctttcgaaagatcccaacgaaaagagagaccacatggtccttcttgagtttgtaacagctgctgggattacacatggcatggatgaactatacaaaccaccaaagccatctgccaagggagccaagaaggccgccaagaccgttacgaagccaaaggacggaaagaagagacgtcatgcccgtaaggaatcatactccgtctacatctaccgtgtcctcaagcaagttcatccagacactggagtttcctccaaagccatgtctatcatgaactcttttgtcaacgatgtcttcgagcgtattgctgctgaagcatcccgtcttgctcactacaacaagcgttccacaatctcatcccgcgaaattcagaccgctgtccgtctgatccttccaggagagcttgccaagcacgccgtgtctgagggaaccaaggccgttaccaagtacacttccagcaagtaggctgtctcatcctactttcacctagttaactgcttgtcttaaaatctatgcttctctttagtatctaaaattttcctagaagcttacaagtatataaatggtctcttctcaataaaggttgtatatttattcatcttattgaatctgccatttcctcgtttttgcgagtttatataccttccaattttctttctattgtattttcaacttctaattttaattcagggaaactgcttcaacgcatcatggaaatcgtctcaagagcatgggaatctgtagatt) generated as described in (Eroglu et al. 2023) was used to insert a *GFP::H2B::SL2* sequence in N2 before *cone-1* start codon.

*cone-1(syb5500)*, *cone-1(syb6898)*, *golg-2(syb6680)*, *golg-4(syb6547)*, *golg-5(syb5400)*, *cone-1(syb5437)*, *cone-1(syb7529)*, *ceh-44(syb7223)*, *ceh-44(syb6281)*, *ceh-44(syb5843)*, *unc-75(syb6499)* were generated by SUNY Biotech.

**Reporter transgenes**

The GFP introduced in all the transgenes and CRISPR insertions in this study have introns.

The *ceh-44 cis-*regulatory element reporters were generated using a PCR fusion approach (Hobert 2002). The *ceh-44prom1* (-2000, -1), *ceh-44prom2* (-26, -1), *ceh-44prom3* (+2075, +2560), *ceh-44prom4* (+4708, +4980), *ceh-44prom5* (+3330, +6689) promoter fragments were amplified from N2 genomic DNA and fused to *2xNLS-GFP*. *ceh-44prom3* and *ceh-44prom4* promoter fragment coordinates match those of the CEH-38/48 ChIP peaks in the regulatory regions of this gene. *ceh-44prom5* contains the entirety of intron 3, exon4, and intron 4 through the last possible in frame start codon on exon 5. The resulting PCR fusion DNA fragments were injected as simple extrachromosomal arrays (50 ng/μL) into *pha-1(e2123)* animals, using a *pha-1* rescuing plasmid (50 ng/μL) as co-injection marker. Extrachromosomal array lines were selected according to standard protocol.

The *unc-75/CELF* rescue line was generated using a rescue plasmid generated in a previous study (Loria et al. 2003), in which an *unc-75* promoter fragment (-2056, -1) drives the expression of human *CELF4*. The plasmid was injected as a simple extrachromosomal array (50 ng/μL) into *unc-75(ot1351); ceh-44(ot1015)* animals (strain OH18463), using an *inx-6prom::tagRFP* plasmid (50 ng/μL) as co-injection marker. Extrachromosomal array lines were selected according to standard protocol.

To rescue the *rab-3* expression defect in the CUT sextuple mutant (strain OH16377, *ceh-38(tm321) II; ceh-44(ot1028) III; ceh-48(tm6112) IV; otIs356 V; otDf1 X*), a rescue plasmid was injected in which a *ceh-48* promoter fragment (-2524, -1876) drives the expression of a *ceh-44* cDNA fragment containing exons 8 to 11. The plasmid was injected as a simple extrachromosomal array (50 ng/μL) into OH16377, using a *ttx-3prom::GFP* plasmid (50 ng/μL) as co-injection marker. Extrachromosomal array lines were selected according to standard protocol.

To rescue the *ric-19* expression defect in *unc-75(ot1351)*, a rescue plasmid was injected in which a *ceh-48* promoter fragment (-2524, -1876) drives the expression of a *ceh-44* cDNA fragment containing exons 5 to 11. The plasmid was injected as a simple extrachromosomal array (50 ng/μL) into *unc-75(ot1351); otIs381(ric-19prom6::2xNLS-GFP)*, using a *inx-6prom::tagRFP* plasmid (50 ng/μL) as co-injection marker. Extrachromosomal array lines were selected according to standard protocol.

The splicing reporter strain (OH18958) was generated using a plasmid produced by Genewiz containing an *eft-3prom::H2B::ceh-44^intron7^::GFP::ceh-44^intron11^::tagRFP* cassette with an *unc-54* 3’UTR. The plasmid was digested with SpeI and injected as a complex extrachromosomal array (0.1 ng/μL) with digested OP50 genomic DNA (100 ng/μL), and a digested *rol-6(su1006)* plasmid (pRF4, 3 ng/μL) as a co-injection marker. Extrachromosomal array lines were selected according to standard protocol.

**Table S1. Strains used in this study.** Promoter coordinates in relation to the ATG.

| **Strain name** | **Strain genotype** | **Source** |
| --- | --- | --- |
| N2 | *C. elegans* Strain N2 (WormBase: WBStrain00000001) | CGC |
| CB950 | *unc-75(e950)* | (Loria et al. 2003) |
| OH10690 | *otIs356(rab-3prom1::2xNLS-tagRFP) V* | (Stefanakis et al. 2015) |
| OH11062 | *otIs381(ric-19prom6::2xNLS-GFP) V* | (Stefanakis et al. 2015) |
| OH16219 | *ceh-44(ot1015[ceh-44::GFP]) III* | (Reilly et al. 2020) |
| OH16377 | *ceh-38(tm321) II; ceh-44(ot1028) III; ceh-48(tm6112) IV; otIs356 V; otDf1 X* | (Leyva-Diaz and Hobert 2022) |
| OH18168 | *ceh-44(ot1015[ceh-44::GFP]), cone-1(ot1282) III* | This study |
| OH18267 | *cone-1(syb5500[cone-1::oxGFP]), golg-4(syb6547[wrmScarlet::golg-4]) III; him-5(e1490) V* | This study |
| OH18268 | *ceh-44(ot1402[ceh-44^ΔUNC-75^]) ot1015[ceh-44::GFP]) III* | This study |
| OH18753 | *unc-75(ot1351) I; otIs381(ric-19prom6::2xNLS-GFP) V* | This study |
| OH18319 | *cone-1(ot1287) III; otIs381(ric-19prom6::2xNLS-GFP) V* | This study |
| OH18347 | *golg-2(syb6680[wrmScarlet::golg-2]) II; cone-1(syb5500[cone-1::oxGFP]) III* | This study |
| OH18418 | *unc-75(e950) I; ceh-44(ot1015[ceh-44::GFP]) III* | This study |
| OH18426 | *otEx8048(ceh-44prom1::2xNLS-GFP, pha-1(+)); pha-1(e2123) III* | This study |
| OH18429 | *otEx8050(ceh-44prom3::2xNLS-GFP, pha-1(+)); pha-1(e2123) III* | This study |
| OH18463 | *unc-75(ot1351) I; ceh-44(ot1015[ceh-44::GFP]) III* | This study |
| OH18465 | *ceh-38(tm321) II; ceh-44(ot1015[ceh-44::GFP]) III; ceh-48(tm6112) IV; otIs356 V; otDf1 X* | This study |
| OH18495 | *otEx8077(ceh-44prom4::2xNLS-GFP, pha-1(+)); pha-1(e2123) III* | This study |
| OH18498 | *otEx8080(ceh-44prom2::2xNLS-GFP, pha-1(+)); pha-1(e2123) III* | This study |
| OH18577 | *cone-1(syb5500[cone-1::oxGFP]) III; pwIs1022[snx-1prom::aman-2::tagRFP]* | This study |
| OH18750 | *cone-1(syb5437[GFP::cone-1] ot1410[cone-1^ΔExon5^]) III* | This study |
| OH18751 | *unc-75(ot1351) I; cone-1(syb5500[cone-1::oxGFP]) III* | This study |
| OH18752 | *unc-75(ot1351) I; cone-1(syb6898[cone-1::T2A::GFP::H2B])* | This study |
| OH18790 | *otEx8142(unc-75prom1::CELF4, inx-6prom::tagRFP);*  *unc-75(ot1351) I; ceh-44(ot1015[ceh-44::GFP]) III* | This study |
| OH18841 | *unc-75(ot1351) I; ceh-44(syb7223[ceh-44^Exon6^::GFP]) I* | This study |
| OH18948 | *ceh-44(ot1447[ceh-44^ΔExon5^] ot1015[ceh-44::GFP]) III* | This study |
| OH18958 | *otEx8170(eft-3prom::H2B::ceh-44intron7::GFP::ceh-44intron11::tagRFP, rol-6(su1006))* | This study |
| OH19071 | *otEx8213(ceh-48prom4::ceh-44(E8-11), ttx-3prom::GFP); ceh-38(tm321) II; ceh-44(ot1028) III; ceh-48(tm6112) IV; otIs356 V; otDf1 X* | This study |
| OH19076 | *otEx8218(ceh-44prom5::2xNLS-GFP, pha-1(+)); pha-1(e2123) III* | This study |
| OH19119 | *cone-1(ot1485[Exon3STOP] syb5500[cone-1::oxGFP]) III* | This study |
| OH19186 | *cone-1(ot1502[GFP::H2B::SL2::cone-1]) III* | This study |
| OH19210 | *ceh-44(ot1486[Exon3STOP] ot1015[ceh-44::GFP]) III* | This study |
| OH19271 | *ceh-44(ot1529[ceh-44^ΔCUT^]) ot1015[ceh-44::GFP]) III* | This study |
| OH19808 | *otEx8302(ceh-48prom4::ceh-44(E5-11), inx-6prom::tagRFP); unc-75(ot1351) I; otIs381(ric-19prom6::2xNLS-GFP) V* | This study |
| PHX5400 | *golg-5(syb5400[golg-5::wrmScarlet]) I* | This study |
| PHX5437 | *cone-1(syb5437[GFP::cone-1]) III* | This study |
| PHX5500 | *cone-1(syb5500[cone-1::oxGFP]) III* | This study |
| PHX5843 | *ceh-44(syb5843[ceh-44^Exon8^::GFP]) III* | This study |
| PHX6281 | *ceh-44(syb6281[ceh-44^Exon7^::GFP]) III* | This study |
| PHX6499 | *unc-75(syb6499[GFP::unc-75]) I* | This study |
| PHX6547 | *golg-4(syb6547[wrmScarlet::golg-4]) III* | This study |
| PHX6680 | *golg-2(syb6680[wrmScarlet::golg-2]) II* | This study |
| PHX6898 | *cone-1(syb6898[cone-1::T2A::GFP::H2B]) III* | This study |
| PHX7223 | *ceh-44(syb7223[ceh-44^Exon6^::GFP]) III* | This study |
| PHX7529 | *cone-1(syb7529[cone-1^Exon4^::GFP]) III* | This study |

**
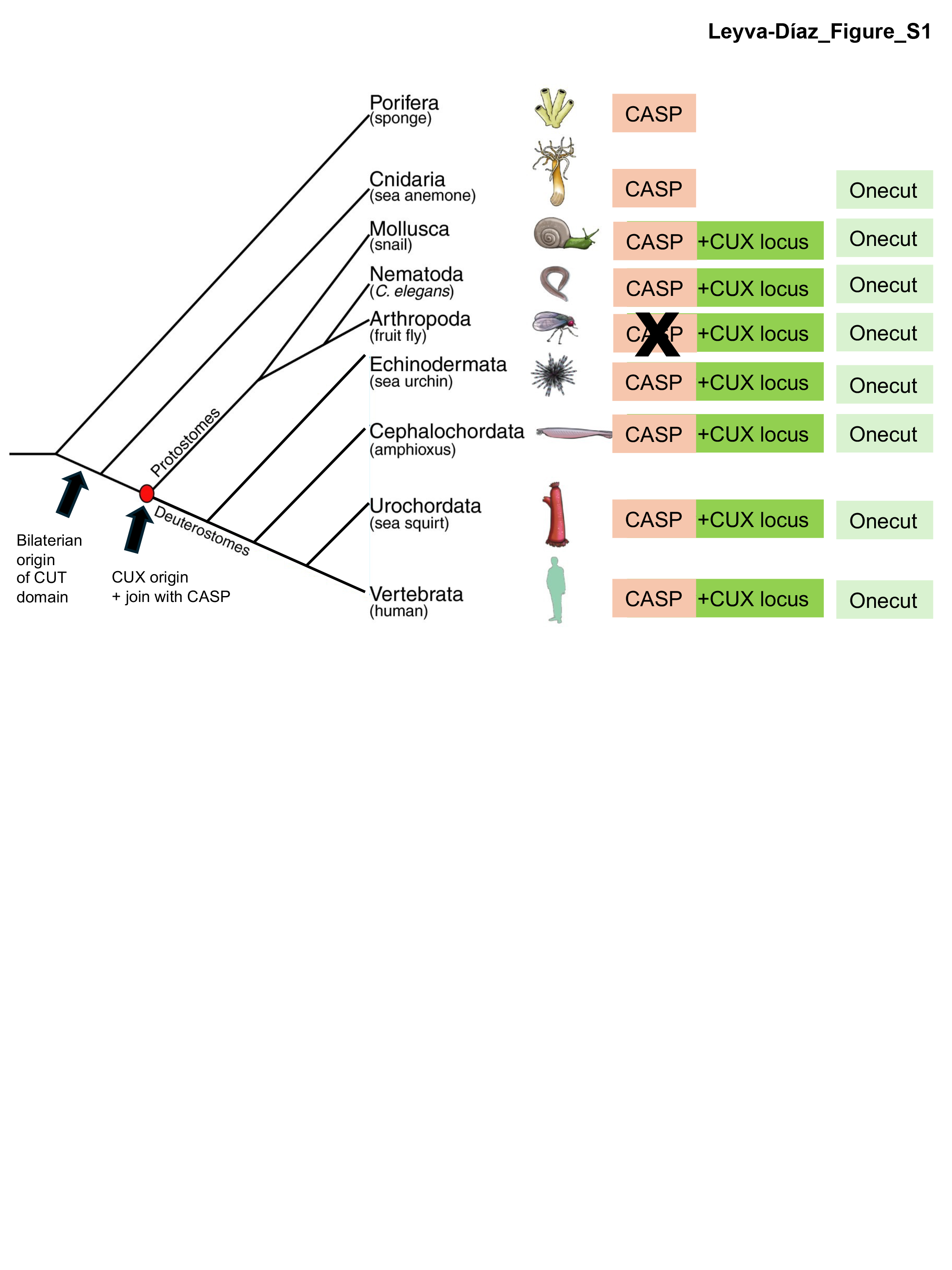
**

**Supplemental Figure S1: Evolutionary history of the CASP/CUX locus.**

The phylogenetic tree with representative species is adapted from (Zhang et al. 2008). The presence of CUX/CASP loci was interrogated by reciprocal BLAST searches using full length CONE-1 and CEH-44/CUX and sequence alignments. Note that CUX and CASP genes do not exist as genomically separate loci. The CUT domain originated at the base of the bilaterians (Brauchle et al. 2018), and the multiplication of the CUT domain apparently coincided with the interposition of the CUX and CASP loci (Burglin and Cassata 2002). The locus structure in urochordates is from *Botrylloides leachii.* The distantly related urochordate *Ciona intestinalis* appears to have lost the CUX1 part of the locus, while it still encodes CASP (A. Stolfi, pers. comm.). Scale bar 10 μm.

**
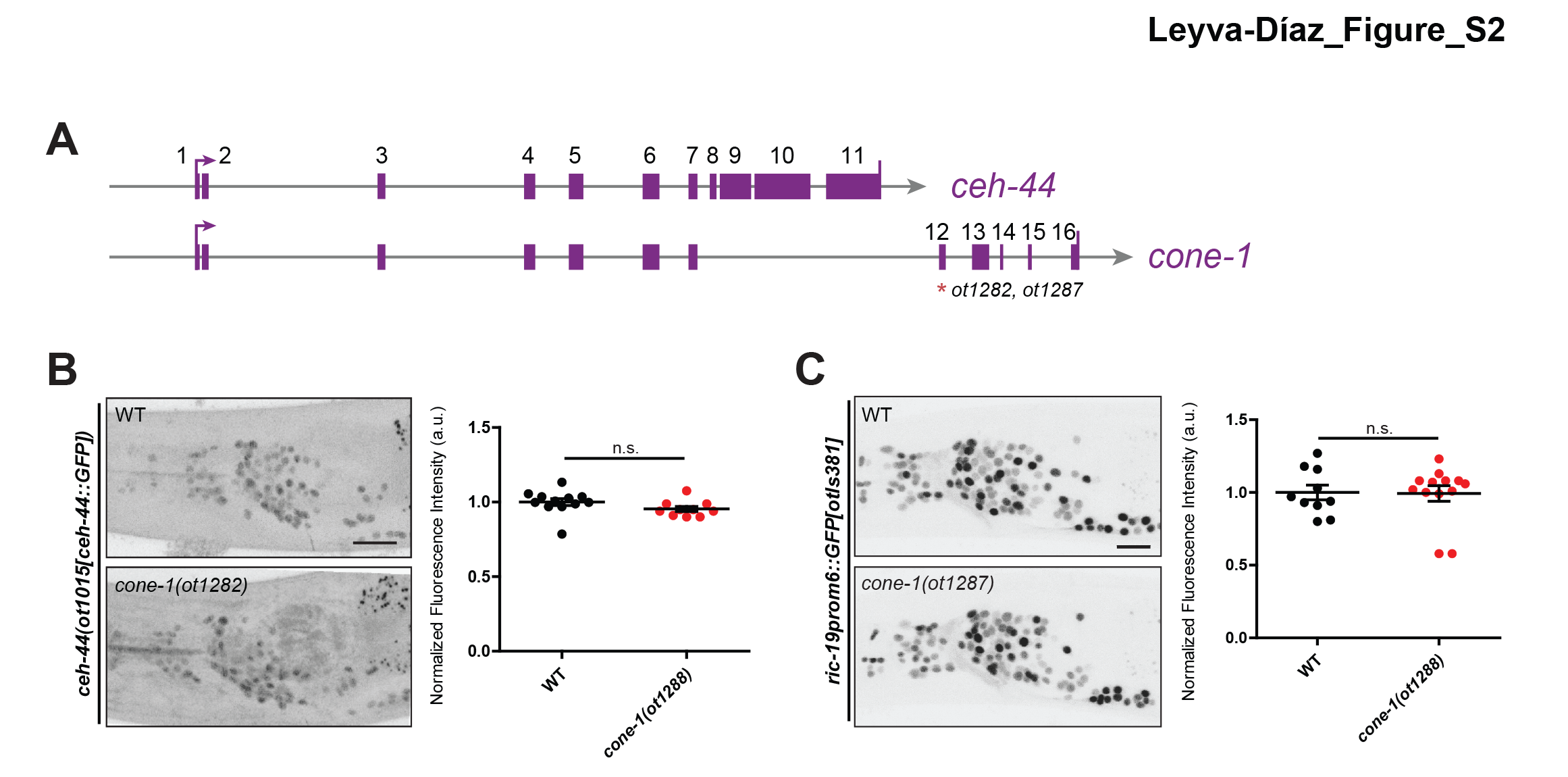
**

**Supplemental Figure S2: *ceh-44/CUX* does not require *cone-1/CASP* for expression or function**

(**A**) Schematic representation of the *cone-1&ceh-44* gene locus showing mutant alleles (red asterisk indicates early stop codon).

(**B-C**) *ceh-44(ot1015[ceh-44::GFP])* (**B**) and *ric19prom6::2xNLS-GFP[otIs381]* (**C**) reporter expression in L4 animals (head, lateral views) in wild-type (top) and *cone-1/CASP* mutants (bottom). Note that the *ot1282* (**B**) and *ot1287* (**C**) mutant alleles are designed to introduce a frameshift and harbor the same molecular lesion. Quantification of fluorescence intensity in head neurons. The data are presented as individual values with each dot representing the expression level of one worm with the mean ± SEM indicated. Unpaired *t*-test, n ≥ 10 for all genotypes.

n.s., not significant; a.u., arbitrary units. Scale bars 10 μm.

**
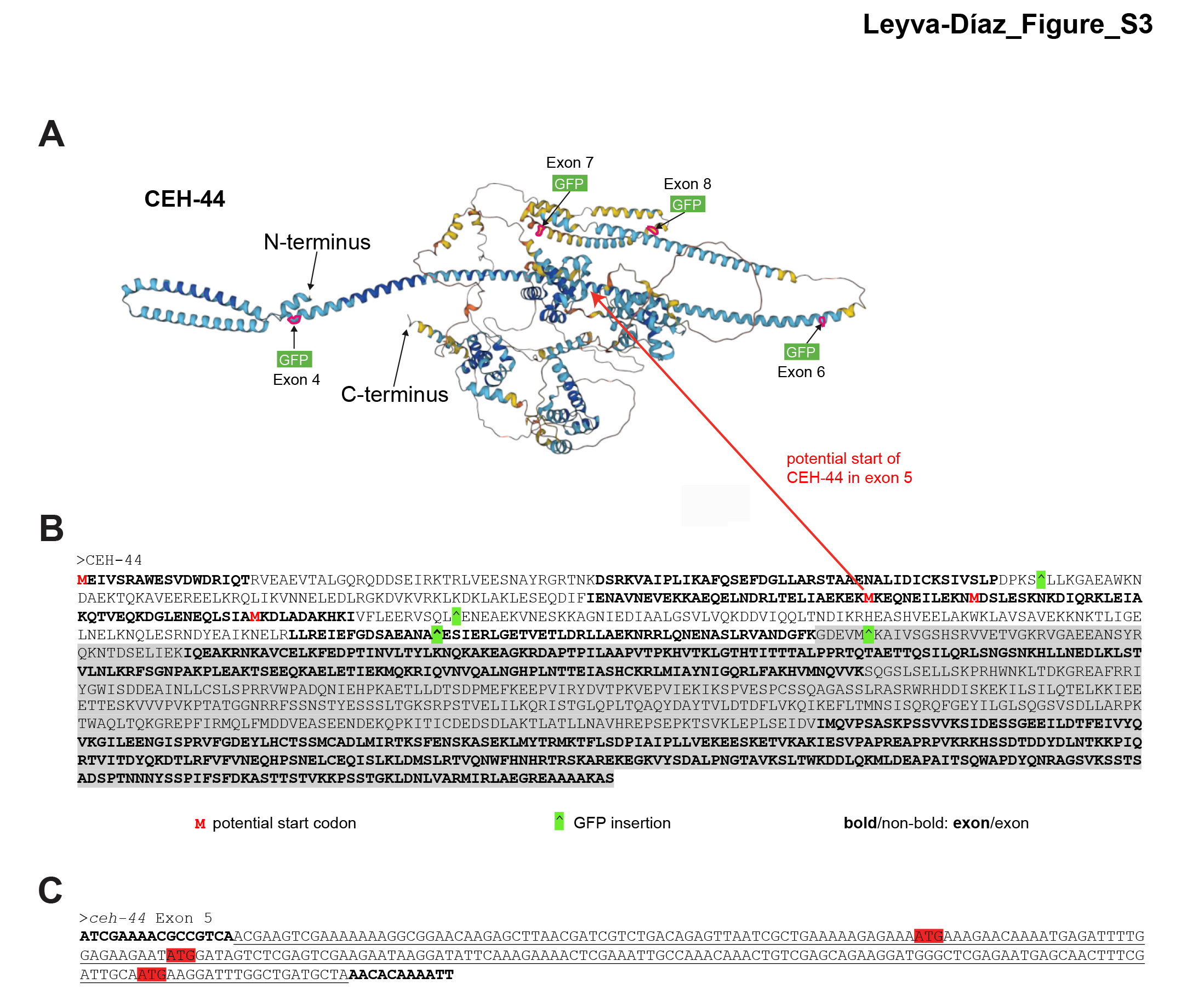
**

**Supplemental Figure S3: GFP insertions throughout *cone-1&ceh-44* locus**

(**A**) AlphaFold (Jumper et al. 2021) prediction of CEH-44 protein structure with GFP insertion locations. Note that the shared part of CEH-44 and CONE-1 is predicted to fold into a large alpha-helical structure. Only a part of this alpha-helical structure is present if the CEH-44 isoform starts at a Methionine in exon 5.

(**B**) CEH-44 protein sequence (Exons 1-11). Residues coded in odd exons are shown in bold, GFP insertion locations depicted in **S3A** are denoted with a caret (^) and highlighted in green, *ceh-44/CUX* specific exons (8-11) are highlighted in grey, methionines within the shared exons (1-7) are shown in red.

(**C**) *ceh-44* exon 5 DNA sequence. Deleted region in *ot1410* and *ot1447* alleles (**Fig. 3A, D, E**) is underlined, and the preserved sequence (in frame) is shown in bold. Three in frame initiation codons are highlighted in red.

**
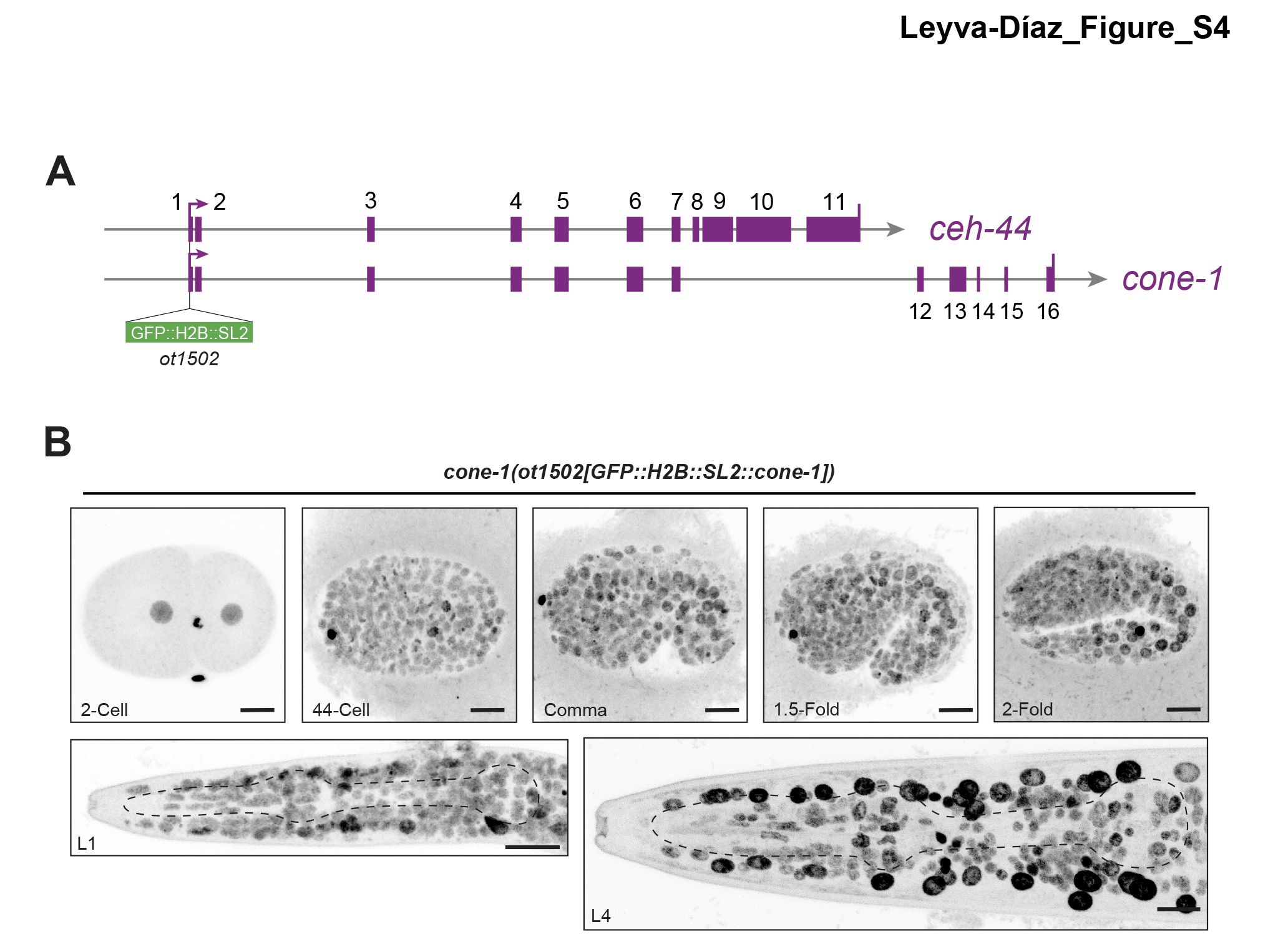
**

**Supplemental Figure S4: Endogenous *cone-1&ceh-44* transcriptional reporter expression**

(**A**) Schematic representation of the *cone-1&ceh-44* gene locus showing the *GFP::H2B::SL2* cassette insertion location.

(**B**) Temporal expression analysis in *cone-1(ot1502[GFP::H2B::SL2::cone-1])* across different embryonic stages (2-Cell, 44-Cell, Comma, 1.5-Fold, 2-Fold) and larval stages (L1 and L4). All images show lateral view. The persistence of GFP::H2B signal over time supports the conclusion that this reflects genuine transcription rather than simply stable histone protein signal. The documented stability of histone reporters usually lasts about 6 hours (Dempsey et al. 2012), but the continued signal indicates ongoing transcription.

Scale bars 10 μm.

**
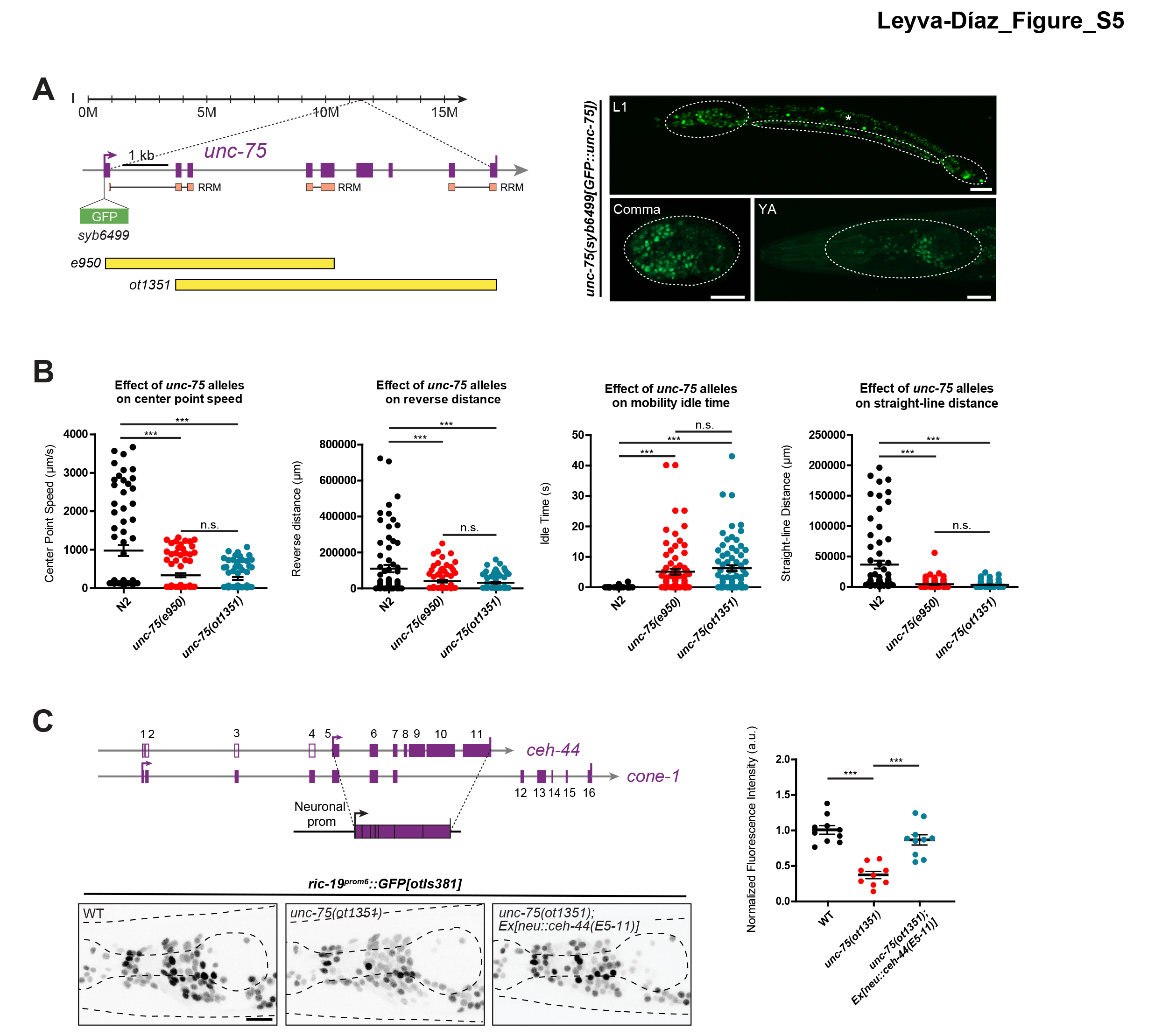
**

**Supplemental Figure S5: Characterization of *unc-75/CELF* expression and function with novel reporter and null alleles.**

(A) Schematic representation of the *unc-75/CELF* gene locus showing the location of the RNA recognition motifs (RRM), GFP insertion and mutant deletion alleles (classic *e950* and *ot1351*, generated in this study*)*. *unc-75(syb6499[GFP::unc-75])* reporter expression shown at the comma embryonic stage (bottom left, lateral view), L1 larval stage (top, full worm lateral view) and young adult stage (bottom right, lateral view of the head). The embryonic comma stage is the stage when neurons are born. Head ganglia, ventral nerve cord, and tail ganglia outlined in L1 image, and head ganglia outlined in young adult image. Asterisk (*) indicate autofluorescence in L1 gut.

(**B**) Worm locomotory features: center point speed (top left), reverse distance (top right), idle time (bottom left), and straight-line distance (bottom right). Each dot represents one worm with the mean ± SEM indicated. Wild-type data are represented with black dots, *unc-75(e950)* with red, and *unc-75(ot1351)* with teal. One-way ANOVA followed by Tukey’s multiple comparisons test; ***p < 0.001. n ≥ 70 for all genotypes.

(**C**) Expression of *ric19prom6::2xNLS-GFP[otIs381]* in wild-type (left), *unc-75(ot1351)* (middle), and *unc-75(ot1351)* mutant rescue (pan-neuronal expression of *ceh-44/CUX* exons 5-11, *neu::ceh-44(E5-11)[otEx8302]*, “neu” = *ceh-48* promoter) (right). Images are lateral head views of L4 animals (max Z-projections). Quantification of fluorescence intensity in head neurons. Each dot represents the expression level within one worm with the mean ± SEM indicated. Wild-type data are represented with black dots, *unc-75(ot1351)* with red dots, and rescue with blue dots. One-way ANOVA followed by Tukey’s multiple comparisons test; ***P < 0.001. n ≥ 10 for all genotypes.

YA, young adult; n.s., not significant; a.u., arbitrary units. Scale bars 10 μm.

**Supplemental Figure S6: Schematic summary of results.** During the proliferative stages of embryogenesis, a primary transcript is generated by upstream regulatory elements active in all cells. This transcript is spliced – through currently unknown mechanisms (“splicing factor X”) – to produce mature CONE-1 protein in all cells. During terminal cellular differentiation, UNC-75 is turned on in all cells of the nervous system, is redirecting splicing to now produce CEH-44 exclusively in neurons. Via intronic regulatory elements several CUT proteins (pan-neuronal CEH-44, pan-neuronal CEH-48 and other ubiquitous CUT proteins) become required to sustain CEH-44 expression in neurons, by promoting the expression of a short and perhaps also the long transcript. This regulatory effect is inferred by (a) the presence of short transcripts (in neurons) and (b) the effect of loss of CUT proteins (or loss of the CUT binding sites) and sustained CEH-44 expression. In non-neuronal cells the ubiquitously produced long transcript continues to be spliced into mature CONE-1 protein (as inferred by the N-terminal fluorophore tagging of CONE-1).

**REFERENCES**

Brauchle M, Bilican A, Eyer C, Bailly X, Martinez P, Ladurner P, Bruggmann R, Sprecher SG. 2018. Xenacoelomorpha Survey Reveals That All 11 Animal Homeobox Gene Classes Were Present in the First Bilaterians. *Genome Biol Evol* **10**: 2205-2217.

Burglin TR, Cassata G. 2002. Loss and gain of domains during evolution of cut superclass homeobox genes. *Int J Dev Biol* **46**: 115-123.

Dempsey WP, Fraser SE, Pantazis P. 2012. PhOTO zebrafish: a transgenic resource for in vivo lineage tracing during development and regeneration. *PLoS One* **7**: e32888.

Dokshin GA, Ghanta KS, Piscopo KM, Mello CC. 2018. Robust Genome Editing with Short Single-Stranded and Long, Partially Single-Stranded DNA Donors in Caenorhabditis elegans. *Genetics* **210**: 781-787.

Eroglu M, Yu B, Derry WB. 2023. Efficient CRISPR/Cas9 mediated large insertions using long single-stranded oligonucleotide donors in C. elegans. *FEBS J* **290**: 4429-4439.

Hobert O. 2002. PCR fusion-based approach to create reporter gene constructs for expression analysis in transgenic C. elegans. *Biotechniques* **32**: 728-730.

Jumper J, Evans R, Pritzel A, Green T, Figurnov M, Ronneberger O, Tunyasuvunakool K, Bates R, Zidek A, Potapenko A et al. 2021. Highly accurate protein structure prediction with AlphaFold. *Nature* **596**: 583-589.

Leyva-Diaz E, Hobert O. 2022. Robust regulatory architecture of pan-neuronal gene expression. *Curr Biol* **32**: 1715-1727 e1718.

Loria PM, Duke A, Rand JB, Hobert O. 2003. Two neuronal, nuclear-localized RNA binding proteins involved in synaptic transmission. *Curr Biol* **13**: 1317-1323.

Reilly MB, Cros C, Varol E, Yemini E, Hobert O. 2020. Unique homeobox codes delineate all the neuron classes of C. elegans. *Nature* **584**: 595-601.

Stefanakis N, Carrera I, Hobert O. 2015. Regulatory Logic of Pan-Neuronal Gene Expression in C. elegans. *Neuron* **87**: 733-750.

Zhang Q, Zmasek CM, Dishaw LJ, Mueller MG, Ye Y, Litman GW, Godzik A. 2008. Novel genes dramatically alter regulatory network topology in amphioxus. *Genome Biol* **9**: R123.
